# Using Carbohydrate Interaction Assays to Reveal Novel Binding Sites in Carbohydrate Active Enzymes

**DOI:** 10.1371/journal.pone.0160112

**Published:** 2016-08-09

**Authors:** Darrell Cockburn, Casper Wilkens, Adiphol Dilokpimol, Hiroyuki Nakai, Anna Lewińska, Maher Abou Hachem, Birte Svensson

**Affiliations:** Enzyme and Protein Chemistry, Department of Systems Biology, Technical University of Denmark, Lyngby, Denmark; Institut National de la Recherche Agronomique, FRANCE

## Abstract

Carbohydrate active enzymes often contain auxiliary binding sites located either on independent domains termed carbohydrate binding modules (CBMs) or as so-called surface binding sites (SBSs) on the catalytic module at a certain distance from the active site. The SBSs are usually critical for the activity of their cognate enzyme, though they are not readily detected in the sequence of a protein, but normally require a crystal structure of a complex for their identification. A variety of methods, including affinity electrophoresis (AE), insoluble polysaccharide pulldown (IPP) and surface plasmon resonance (SPR) have been used to study auxiliary binding sites. These techniques are complementary as AE allows monitoring of binding to soluble polysaccharides, IPP to insoluble polysaccharides and SPR to oligosaccharides. Here we show that these methods are useful not only for analyzing known binding sites, but also for identifying new ones, even without structural data available. We further verify the chosen assays discriminate between known SBS/CBM containing enzymes and negative controls. Altogether 35 enzymes are screened for the presence of SBSs or CBMs and several novel binding sites are identified, including the first SBS ever reported in a cellulase. This work demonstrates that combinations of these methods can be used as a part of routine enzyme characterization to identify new binding sites and advance the study of SBSs and CBMs, allowing them to be detected in the absence of structural data.

## Introduction

Carbohydrates exist in many forms in nature, from the *O*- and *N*-linked glycans of proteins to the cell walls of plants, fungi and bacteria as well as storage polymers such as starch and glycogen. Enzymes active towards these carbohydrates must have the means to identify and act on their particular substrate within an often complex milieu. A common strategy for accomplishing this recognition is to utilize binding regions outside of the active site that are less constrained as they need not bind the substrate in an optimal mode for catalysis. These non-catalytic binding sites can operate in conjunction with the active site in catalysis, or may simply serve to localize the enzymes to the substrates. Such external sites interacting with carbohydrates exist on a continuum from independent domains, termed carbohydrate binding modules (CBMs) [1], to regions or spots termed surface binding sites (SBSs) situated on the catalytic domain at a certain distance from the active site [2–4]. Enzymes can contain multiple CBMs, multiple SBSs or combinations of both and these carbohydrate binding sites can perform diverse functions related to enzyme activity [2]. The importance of CBMs has long been established, particularly for enzymes acting on insoluble polysaccharides, and they are formally organized into families in the CAZy database [5,6] (www.cazy.org). SBSs, however, are much less studied and also much less frequently occurring, with the majority of the investigated enzymes participating in starch or xylan degradation. Thus of the approximately 60 enzymes from 20 CAZy families to date identified to possess one or more SBSs, almost half belong to GH13, the α-amylase family. It is likely, however, that since SBSs have commonly been detected only by observation in crystal structures of ligand complexes, they are in fact more widespread among the CAZy families and have to now gone unappreciated.

A variety of methods have been used to study the characteristics of SBSs and CBMs, including isothermal titration calorimetry (ITC) [7,8], surface plasmon resonance (SPR) [9–11], insoluble polysaccharide pulldown (IPP) [9,10,12], affinity electrophoresis (AE) [9,13], confocal laser scanning microscopy (CLSM) [9,14] and enzyme activity progress curves [15]. This set of assays enables measurement of binding properties of known sites, AE and CLSM having been applied to qualitatively demonstrate binding; although AE can also be used to quantify the affinity [13]. Mutational analysis has been used to confirm the presence and location of the binding site and disclose its impact on enzyme activity [10,16–19]. Many sequences suspected to perform binding functions within carbohydrate active enzymes remain uncharacterized and there are probably many more that are unsuspected. Importantly, just as IPP, AE and SPR are useful to describe the known binding sites, in this work we show these techniques are suitable for revealing the presence of and to further characterize hitherto unknown SBSs as well as CBMs, even in the absence of a solved crystal structure.

## Materials and Methods

### Enzymes

The investigated carbohydrate active enzymes are designated by their CAZy family (www.cazy.org), followed by a number to separate these cases where more than one enzyme from a particular family were studied. The identification, properties and origins of the enzymes, which either stemmed from our research group, were donated by other researchers or were commercially available are listed in Table 1. GH5-1 [20], GH13-1 [21], GH13-2 [22], GH15-1/GH15-2 [23], GH36-1 [24], GH62-1 [18], GH65-1 [25] and GH94-1/GH94-2 [26] were produced in-house as previously described. AA09-1, GH10-1 and GH11-1 were cloned from *Aspergillus nidulans* FGSC A4 cDNA [27], and GH32-1 and GH32-2 from *Lactobacillus acidophilus* NCFM genomic DNA (primer pairs are listed in S1 Table). See the Supplementary Methods section (S1 File) for the full details of the expression and purification protocols.

**Table 1:** Origins and binding properties of enzymes in this study.

ID	Activity	Organism	CBM	AE Binding^h^	IPP^h^
AA9-1^a^	Lytic polysaccharide monoxygenase	*Aspergillus nidulans*	No CBM	None	None
CE2-1^b^	Acetyl xylan esterase	*Cellvibrio japonicus*	No CBM	None	None
GH1-1^b^	β-glucosidase	*Rhizobium etli*	No CBM	None	None
GH3-1^c^	Exo-glucanase	*Hordeum vulgare*	No CBM	None	None
GH5-1^a^	Endo-β-1,4-mannanase	*Aspergillus nidulans*	No CBM	KGM	None
GH6-1^d^	Endoglucanase	*Thermobifida fusca*	No CBM	HEC, KGM, LAM, XYG	None
GH8-1^e^	Endoglucanase	*Clostridium thermocellum*	No CBM	HEC, KGM, WAX	None
GH10-1^a^	Xylanase	*Aspergillus nidulans*	No CBM	XYG	None
GH11-1^a^	Xylanase	*Aspergillus nidulans*	No CBM	BWX, LAM, PUL, OSX, WAX	None
GH13-1^a^	α-amylase	*Hordeum vulgare*	No CBM	PUL, AP, GLY	STA
GH13-2^a^	Limit dextrinase	*Hordeum vulgare*	CBM48	AM	None
GH13-3^b^	α-amylase	*Escherichia coli*	No CBM	NT^i^	CHI
GH13-4^b^	Isoamylase	*Escherichia coli*	No CBM	None	NT^i^
GH13-5^b^	Pullulanase	*Bacillus subtilis*	CBM48	NT^i^	None
GH13-6^f^	α-amylase	*Sus scrofa*	No CBM	HEC, HA, AM, GAL, ARA, PUL, GLY	None
GH14-1^f^	β-amylase	*Hordeum vulgare*	No CBM	None	OSX
GH15-1^a^	Glucoamylase	*Aspergillus niger*	CBM20	AM, GAL, PUL, AP, GLY	STA
GH15-2^a^	Glucoamylase	*Aspergillus niger*	No CBM	None	None
GH26-1^b^	β-mannanase	*Cellvibrio japonicus*	No CBM	None	None
GH27-1^b^	α-galactosidase	*Clostridium cellulolyticum*	CBM6	NT^i^	None
GH31-1^f^	α-glucosidase	*Oryza sativa*	No CBM	RHG, KGM, BWX, GAL, LAM, XYG	CHI, OSX
GH32-1^a^	β-fructosidase	*Lactobacillus acidophilus*	No CBM	None	None
GH32-2^a^	Sucrose-6-phosphate hydrolase	*Lactobacillus acidophilus*	No CBM	None	None
GH36-1^a^	α-galactosidase	*Aspergillus nidulans*	No CBM	None	None
GH43-1^b^	β-xylosidase	*Bacillus subtilus*	No CBM	None	None
GH48-1^e^	Processive endoglucanase	*Clostridium cellulolyticum*	No CBM	RHG, KGM, BBG, WAX	CEL
GH53-1^b^	β-galactanase	*Cellvibrio japonicus*	No CBM	None	STA
GH62-1^a^	α-L-arabinofuranosidase	*Aspergillus nidulans*	No CBM	HEC, KGM, BWX, BBG, OSX, WAX	None
GH65-1^a^	Maltose phosphorylase	*Lactobacillus acidophilus*	No CBM	None	None
GH77-1^g^	Amylomaltase	*Escherichia coli*	No CBM	None	CEL, STA
GH84-1^b^	O-GlcNAcase	*Streptococcus pyogenes*	No CBM	KGM	None
GH85-1^b^	Endo-β-*N*-acetylglucosaminidase	*Clostridium perfringens*	No CBM	None	None
GH94-1^a^	Cellobiose phosphorylase	*Clostridium thermocellum*	No CBM	None	None
GH94-2^a^	Cellodextrin phosphorylase	*Clostridium thermocellum*	No CBM	None	None
PL10-1^b^	Pectate lyase	*Caulobacter crescentus*	No CBM	None	STA

^a^ produced as previously described (see materials and methods)

^b^ from Prozomix (Haltwhistle, United Kingdom)

^c^ from Maria Hrmova (University of Adelaide)

^d^ from David Wilson (Cornell University)

^e^ from NZYtech (Lisbon, Portugal)

^f^ from Sigma

^g^ from Alison Smith (John Innes Center)

^h^ see Table 2 for polysaccharide identities

^i^ Not tested

### Affinity Electrophoresis

AE involves two native polyacrylamide gel electrophoresis (PAGE) experiments run together. One conventional native PAGE gauges the regular position of the protein of interest, while in a second the polysaccharide under investigation is incorporated when casting the polyacrylamide gel. Proteins migrating slower in the second gel as compared to the control are presumed to interact with the polysaccharide. To account for variation in gel pore size due to polysaccharide incorporation, reference proteins are included in both gels. The stained gels were scanned and then analyzed using the program ImageJ (http://imagej.nih.gov/ij/). For the target enzyme, the ratio of the migration distances in the presence of polysaccharide to that in the control gel was divided by the corresponding ratio for a reference protein chosen from the molecular weight marker to migrate closest in the control gel to the target enzyme. Thus proteins not binding the polysaccharide have ratios of approx. 1, while those binding to polysaccharides have ratios < 1. In the present study, ratios < 0.85 were considered positive for binding to account for average band thickness and average migration distance to allow stringent hypothesis testing, minimizing false positives. Most polysaccharides were used at a final concentration of 0.1%; although some were used at 0.05% due to limited solubility and/or changes in gel properties at higher concentrations (see Table 2). Proteins were analyzed in one of four regimes of conditions depending on size and isoelectric point (Table 3), all gels being buffered by 50 mM Tris-borate pH 8.8 and run at 4°C. For the enzymes active on cellulose: GH6-1, GH8-1 and GH48-1, the effect of 100 mM cellobiose was tested by including it in the gels, loading and running buffers. The migration in control, hydroxyethyl cellulose (HEC) or barley β-glucan (BBG) containing gels were compared to runs in corresponding gels without cellobiose. All of these gels were run according to the AE3 conditions (Table 3).

**Table 2:** Properties of polysaccharides used in this study.

Polysaccharide^a^	Monosaccharide Units	Linkage
Amylopectin (AP, 0.1%)	Glucose	α1–4 (backbone), α-1,6 (branch points)
Amylose (AM, 0.05%)	Glucose	α1–4
Avicel (crystalline cellulose, CEL)	Glucose	β1–4
Barley β-glucan (BBG, 0.1%)	Glucose	Mixture of β1–3 and β1–4
Birchwood xylan (BWX, 0.1%)	Xylose	β1–4
Chitin (CHI)	*N*-acetylglucosamine	β1–4
Curdlan (CLN, 0.05%)	Glucose	β1–3
Galactomannan (GLM, 0.05%)	Mannose, Galactose	β1–4 mannose backbone, α1–6 galactose sidechains
Glycogen (GLY, 0.1%)	Glucose	α1–4 (backbone), α1–6 (branch points)
Hyaluronic acid (HA, 0.1%)	Glucuronic acid, *N*-acetylglucosamine	β1–4 and β1–3
Hydroxyethyl cellulose (HEC, 0.1%)	Glucose	β1–4 with CH_2_CH_2_OH at O2, O3 or O6
Konjac glucomannan (KGM, 0.1%)	Mannose, Glucose	Mixture of β1–4 linked mannose and glucose
Laminarin (LAM, 0.1%)	Glucose	β1–3 interspersed with some β1–6
Oat spelt xylan (OSX, 0.1%)	Xylose, Arabinose, Glucose	β1–4 xylose backbone, α1–2 or α1–3 arabinose or glucose branches
Potato galactan (GAL, 0.05%)	Galactose	β1–4
Pullulan (PUL, 0.05%)	Glucose	(α1–4)_3_ repeat linked by α1–6
Rhamnogalacturonan I (RHG, 0.1%)	Galacturonic acid, Rhamnose, others	α1–2 and α1–4, others for sidechains
Starch (STA)	Glucose	α1–4 (backbone), α1–6 (branch points)
Sugar beet arabinan (ARA, 0.1%)	Arabinose	α1–5
Wheat arabinoxylan (WAX, 0.1%)	Xylose, Arabinose	β1–4 xylose backbone, α1–2 or α1–3 arabinose branches
Xyloglucan (XYG, 0.1%)	Xylose, Glucose	β1–4 glucose, α1–6 xylose sidechains

^a^ in brackets are the abbreviations used in this study and the concentration used in AE

**Table 3:** Affinity electrophoresis regimes.

Regime	Acrylamide conc. (%)	Potential (V)	Time (h)	Enzymes
AE1	6.5	80	4.5	GH3-1, GH11-1, GH13-2, GH14-1, GH31-1, GH32-2, GH36-1, GH65-1, GH77-1, GH94-1, GH94-2
AE2	12.0	45	16.0	AA9-1, GH5-1, GH6-1, GH10-1, GH13-1, GH13-6, GH15-1, GH15-2, GH32-1, GH62-1
AE3	12.0	50	18.0	GH1-1, GH8-1, GH13-4, GH26-1, GH43-1, GH48-1, GH85-1
AE4	6.5	80	6.0	CE2-1, GH3-1, GH53-1, GH84-1, PL10-1

### Insoluble Polysaccharide Pulldown

IPP was performed for avicel (approx. 50% crystalline cellulose), corn starch, chitin and oatspelt xylan. Polysaccharide (5 mg) in a 1.5 mL Eppendorf tube, was washed three times with 10 mM HEPES pH 7.0, 150 mM NaCl followed by addition of enzymes (200 μL, 0.2 mg/mL) and incubated (1 h, 4°C) with agitation. After pelleting (20 000 x g, 10 min, 4°C) insoluble polysaccharides and bound enzymes, the supernatants were decanted and centrifuged again to remove trace polysaccharides, followed by protein concentration determination (BioRad, Hercules, California; based on the Bradford method [28]). The fraction of protein bound was calculated by dividing the final protein concentration by the initial protein concentration. For the GH6-1, GH8-1 and GH48-1 enzymes active on cellulose, the effect of 100 mM cellobiose or 4 mM cellopentaose on their binding to avicel was also measured (in duplicate).

### Surface Plasmon Resonance

The affinity and binding stoichiometry for oligosaccharides of the cellulases GH6-1, GH8-1 and GH48-1 and the GH13 enzymes GH13-3, GH13-5 and GH13-6 were analyzed using SPR (Biacore T100; GE Healthcare). The cellulases were immobilized on CM5 chips (GE Healthcare) *via* amine coupling to carboxyl groups on the chip according to the manufacturer’s protocol, while the GH13 enzymes were biotinylated and immobilized on streptavidin chips as previously described [17]. Some GH13 enzymes are not able to withstand the low pH used for the immobilization on CM5 chips and thus the alternative immobilization on streptavidin was used, though this requires somewhat more protein. Enzymes were immobilized at 1500 response units (RU) in the presence of 5 mM of an anticipated ligand, i.e. cellopentaose for cellulose active enzymes and β-cyclodextrin in the case of starch active enzymes to protect binding sites. The immobilized enzymes were assayed for binding to 10 — 5000 μM cellopentaose, xylotetraose and maltotetraose for the cellulases or β-cyclodextrin, maltohexaose and a DP10 limit dextrin (in house prepared as in Roberts and Whelan [29]) for the GH13 amylolytic enzymes. Stoichiometry was calculated using the formula: $S=\frac{MW_{L}}{MW_{E}}*\;\frac{R_{E}}{R_{max}}$ where S is the stoichiometry, MW_L_ is the molecular weight of the ligand, MW_E_ is the molecular weight of the enzyme, R_E_ is the number of response units of enzyme bound to the chip and R_max_ is the maximum response calculated from a one site binding model fitted to the data using the Biacore software.

### Surface Binding Site Conservation Analysis

For enzymes that have a related family member known to possess an SBS, conservation of the residues comprising the SBS was examined by sequence alignment. The enzymes from this study and the PDB ID of the corresponding family member are: GH1-1 (1UYQ, 1GNX), GH5-1 (2PC8), GH8-1 (2B4F), GH10-1 (1GOQ, 1B3V), GH11-1 (2QZ2, 2QZ3), GH13-3 (2D3N), GH14-1 (1B9Z), GH15-1 (2F6D), GH27-1 (2HG2) and GH31-1 (3POC). Residues involved in forming the SBSs were identified in consultation with the literature and by manual inspection of the crystal structures using PyMol. Alignments were performed in CLC Sequence viewer v7 (CLC Bio) using the default algorithm.

## Results

### Properties of Enzymes Studied

Thirty five enzymes from 27 different families in the CAZy database [6] were examined for the possible presence of an SBS (see Table 1). The vast majority are glycoside hydrolases, but glycosyl transferases, lytic polysaccharide monoxygenases, carbohydrate esterases, polysaccharide lyases and carbohydrate phosphorylases are also represented. Most of the enzymes are from bacteria or fungi, some are from plants and one is of mammalian origin. They were chosen to embody large diversity and to have a few characteristics facilitating their study by the chosen techniques. First, most enzymes contained no CBM, which simplifies interpretation of the binding data. In case of the glucoamylase from *Aspergillus niger* forms with and without its native CBM20 were compared. Secondly, three enzymes known to have SBS(s),barley α-amylase AMY1 (GH13-1) [17,30,31], pig pancreatic α-amylase (GH13-6) [32,33] and pullulanase from *Bacillus subtilis* (GH13-5; PDB ID: 2E9B, unpublished), were selected as positive controls in the screening process. Additionally, three thoroughly structurally characterized enzymes in complex with carbohydrate ligands without a detected SBS served as pseudo-negative controls, although we cannot definitively exclude the possibility that they possess an SBS. These were barley β-amylase (GH14-1) [34,35], *Cellvibrio japonicus* β-mannanase (GH26-1) [36–39] and *Clostridium cellulolyticus* processive endoglucanase (GH48-1) [40–42]. Finally, all but one (GH94-2) of the tested enzymes have molecular mass < 100 kDa and all had an isoelectric point < pH 7, compatible with AE in the four applied regimes. It should be emphasized that these are not hard limits for the technique. Thus for higher molecular weights longer run times can be used, while for higher pI, a lower buffer pH can be used and the electrodes switched to allow migration of positively charged proteins.

### Affinity Electrophoresis

**Fig 1:**
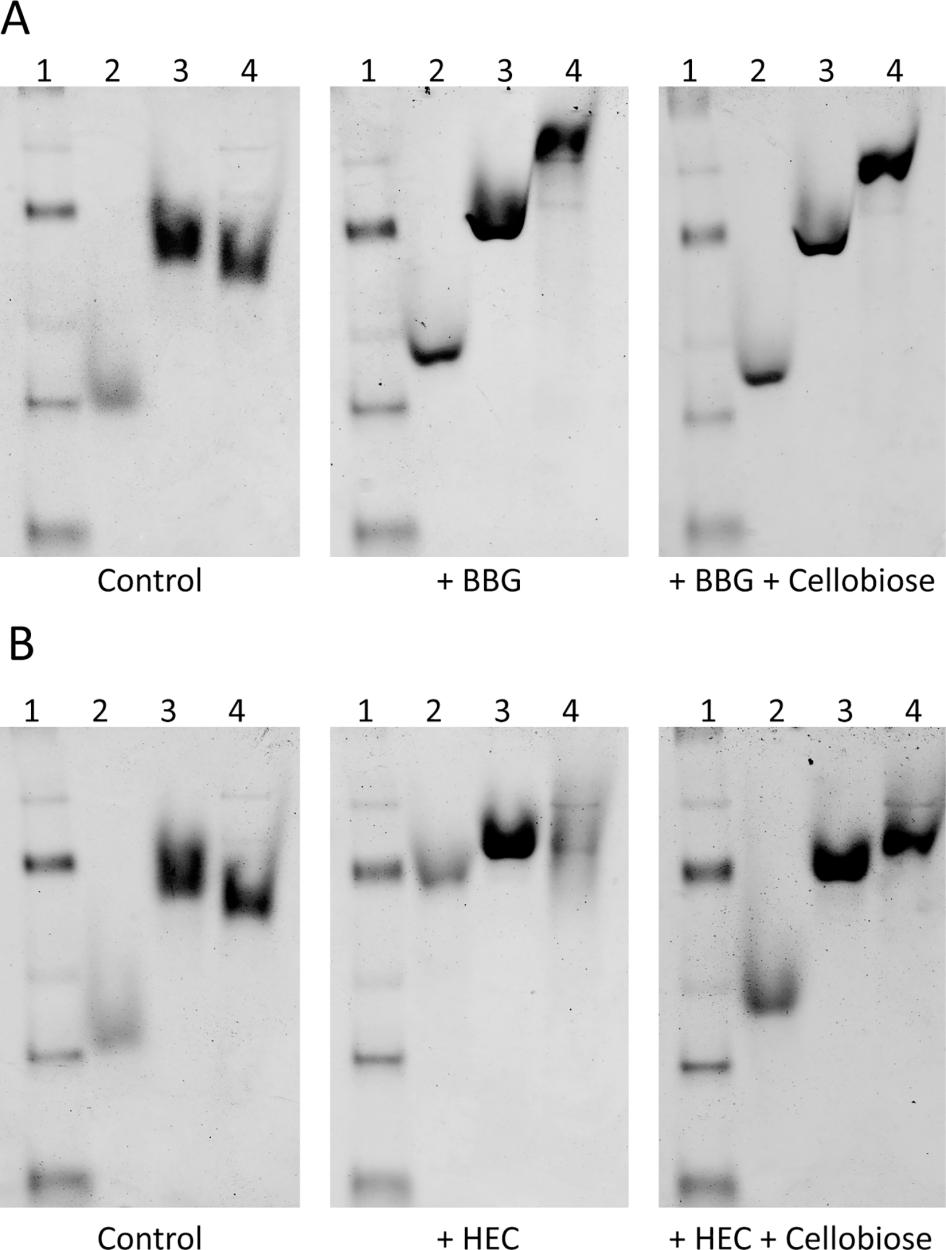
Affinity electrophoresis of cellulases with and without cellobiose. AE was performed with the polysaccharides barley β-glucan (A; BBG) and hydroxyethyl cellulose (B; HEC) in the presence and absence of the active site inhibitor cellobiose. Lane 1 is a protein ladder (NativeMark from Invitrogen), lane 2 is GH6-1, lane 3 GH8-1 and lane 4 GH48-1.

Thirty five enzymes were tested for interactions through AE with 18 polysaccharides (Table 1), providing a variety of monosaccharide units and linkages (summarized in Table 2) representing carbohydrate structures relevant to the enzymes under study. Three of the 35 enzymes appeared unsuitable for AE as they did not give a distinct band in the native PAGE. These were not screened against the full set of polysaccharides, but still by IPP. Of the 18 polysaccharides, only galactomannan and curdlan do not detectably interact with any of the 32 enzymes. By contrast konjac glucomannan very interestingly retards seven different enzymes in AE, only three of which (GH5-1, GH6-1, GH8-1) are expected to have activity against glucomannan. Noticeably, glucomannan does not retard GH26-1 even though it is a substrate [39]. Nineteen of the enzymes show no significant binding with any of the polysaccharides in AE (Table 1). Of the 13 enzymes that interact with a polysaccharide in AE, nine bind to several (from three to seven) polysaccharides, often of diverse linkage type and/or monosaccharide composition. For instance GH13-6 (pig pancreas α-amylase) interacts with the starch related α-glucans glycogen, amylose and pullulan as expected, (whereas amylopectin was just outside the 0.85 polysaccharide:control ratio chosen as the cutoff indicative of binding), plus with other polysaccharides as diverse as cellulose, hyaluronic acid, arabinan and galactan. Also the α-glucosidase GH31-1 interacts with several different polysaccharides, none of these, however, are starch related. Some of the screened enzymes thus appear surprisingly promiscuous in their affinity for polysaccharides. To further investigate the utility of AE to characterize SBSs, three cellulases were tested with BBG and HEC in the presence and absence of the active site inhibitor cellobiose (Fig 1). The added cellobiose clearly competes with HEC for binding to GH6-1 and GH8-1, whereas it only slightly weakens the binding of GH48-01 to BBG.

### Insoluble Polysaccharide Pulldown

**Fig 2:**
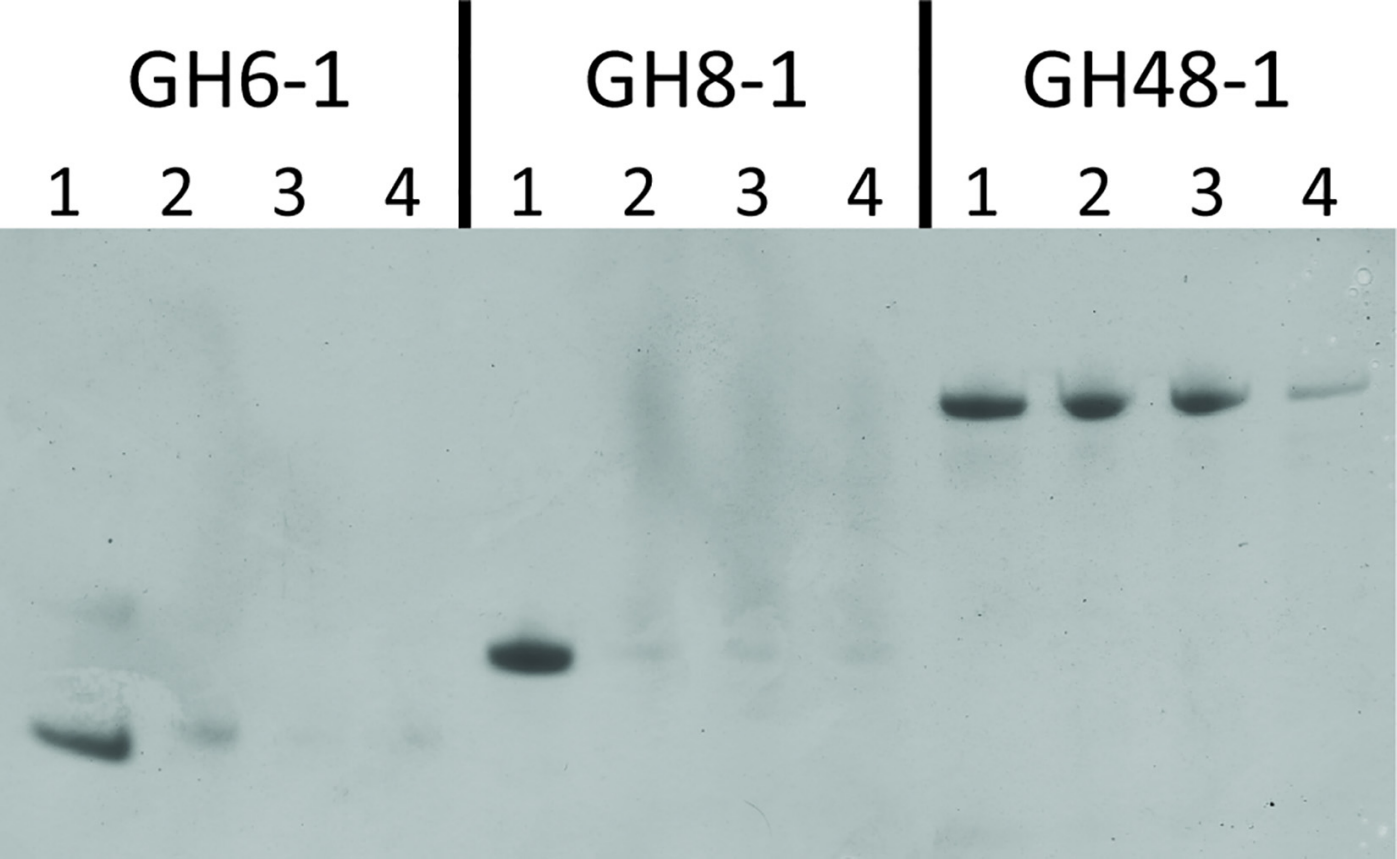
SDS-PAGE of fractions from insoluble polysaccharide pulldown assays of the cellulases GH6-1, GH8-1 and GH48-1 with avicel. For each protein lane 1 is the initial protein sample, lane 2 the fraction bound to avicel in the absence of inhibitors, lane 3 the fraction bound to avicel in the presence of 100 mM cellobiose and lane 4 the fraction bound to avicel in the presence of 4 mM cellopentaose.

IPP was performed as a complement to AE that monitors interactions with soluble polysaccharides. A total of 34 enzymes (1 produced inconsistent results between replicates and is not included) were tested with four carbohydrates; cellulose, insoluble starch, chitin and xylan (Table 1). The positive controls barley α-amylase (GH13-01) and glucoamylase (GH15-1; containing a starch specific CBM20) as expected bind to insoluble starch. Overall 7 out of 34 enzymes tested bind to one and 2 out of 34 enzymbes bind to two insoluble polysaccharides, respectively. As with AE, promiscuous binding occurs. Most remarkably the α-glucosidase GH31-1 binds to two non-starch compounds, but not to starch itself. The three cellulases GH6-1, GH8-1 and GH48-1 were again examined in more depth for interaction with cellulose in the presence and absence of either cellobiose or cellopentaose (Fig 2), which both bind to their active sites. Remarkably, even in the absence of these competitive ligands neither GH6-1 nor GH8-1 interact strongly with avicel, but exhibit weak binding that for GH6-1 is inhibited by cellobiose and cellopentaose, while GH8-1 binding is unaffected by these oligosaccharides. Remarkably, GH48-1, binds tightly to avicel, which is not changed by cellobiose, but efficiently reduced by cellopentaose.

### Surface Plasmon Resonance

AE and IPP are useful for detecting binding to soluble and insoluble polysaccharides, respectively, however, they cannot provide information about the number of binding sites. SPR conversely both monitors the affinity and quantifies the bound carbohydrate, thus revealing an apparent binding stoichiometry. To examine the utility of SPR for uncovering multiple binding sites, three GH13 family enzymes (GH13-3, GH13-5 and GH13-6) and three cellulases (GH6-1, GH8-1 and GH48-1) are analyzed for affinity and stoichiometry using different oligosaccharides (Table 4). With the GH13 enzymes are used: β-cyclodextrin (a cyclic form of maltoheptaose), maltohexaose and an α-limit dextrin (a DP10 maltooligosaccharide containing an α-1,6 branch point). Cellopentaose is tested with the cellulases and compared to the distinctly different maltotetraose and xylotetraose to probe not only the affinity, but also the specificity of recognition, given the promiscuous interactions seen via the other assays. GH13-5 (PDB ID: 2E9B, unpublished) and GH13-6 [32,33] possess SBSs as seen in crystal structures. All three GH13 enzymes give stoichiometries approaching 2 for β-cyclodextrin (Table 4), indicating the presence of at least one and possibly more SBSs, since in some GH13 enzymes β-cyclodextrin is not bound at the active site [10]. The measured fractional stoichiometry can arise because the enzymes are immobilized on the chip in different orientations, not having all binding sites well exposed [9]. Thus binding stoichiometry estimates are typically rounded up to give meaningful numbers. The *E*. *coli* α-amylase (GH13-3) and *B*. *subtilis* pullulanase (GH13-5) interact weakly with maltohexaose and α-limit dextrin, although maltohexaose is a substrate for the former [43] and α-limit dextrin for the latter enzyme [44]. In neither case were kinetics analyzed for these enzyme/substrate combinations and they are probably poor substrates. For porcine pancreatic α-amylase (GH13-6), the stoichiometry with maltohexaose is > 3 (Table 4) implying the presence of four different binding sites. This fits well with the crystal structure in complex with maltopentaose accommodated at the active site and three SBSs [33]. Notably, as a one-site binding model (not shown) fits well to the SPR data as the affinities of the individual sites are not differentiated. The binding stoichiometry of α-limit dextrin with GH13-6 is close to 1, suggesting the α1—6 linked branch in the maltooligosaccharide suppresses binding at the several SBSs known to bind linear maltooligosaccharides [33]. The cellulases GH6-1 and GH48-1 give a stoichiometry with cellopentaose below 1, probably reflecting the absence of multiple binding sites and that this interaction takes place at the active site. However, the stoichiometry of 2.3 for cellopentaose binding to the GH8-1 enzyme identifies three sites conceivably encompassing two SBSs. As expected the three cellulases show no significant affinity for maltotetraose and xylotetraose, although GH8-1 interacts weakly with these oligosaccharides.

**Table 4:** Binding affinities and stoichiometries for family GH13 enzymes and cellulases as determined by surface plasmon resonance.

**Enzyme**	**β-cyclodextrin**		**Maltohexaose**		**α-Limit Dextrin**	
	***K*_d_ (mM)**	**Stoichiometry**	***K*_d_ (mM)**	**Stoichiometry**	***K*_d_ (mM)**	**Stoichiometry**
GH13-3	1.36 ± 0.05	1.63 ± 0.13	NB^a^		NS^b^	
GH13-5	0.97 ± 0.13	1.51 ± 0.20	NB^a^		NB^a^	
GH13-6	0.87 ± 0.40	1.69 ± 0.32	1.33 ± 0.12	3.23 ± 0.13	1.91 ± 0.22	0.88 ± 0.09
**Enzyme**	**Cellopentaose**		**Maltotetraose**		**Xylotetraose**	
	***K***_**d**_ **(mM)**	**Stoichiometry**	***K***_**d**_ **(mM)**	**Stoichiometry**	***K***_**d**_ **(mM)**	**Stoichiometry**
GH6-1	0.33 ± 0.11	0.20 ± 0.03	NB^a^		NB^a^	
GH8-1	0.86 ± 0.09	2.33 ± 0.55	NS^b^		NS^b^	
GH48-1	2.07 ± 0.78	0.85 ± 0.42	NB^a^		NB^a^	

^a^ No binding detected.

^b^ Binding was not saturable under the conditions tested.

## Discussion

### Screening Strategy

In this study we have started out with a reasonably large collection of 35 enzymes and screened these for binding to 18 soluble and 4 insoluble polysaccharides using AE and IPP, respectively. These techniques have several advantages. First, they can be performed in a relatively high throughput manner, so numerous enzyme-polysaccharide interactions can be examined in a relatively short time. Second, they are inexpensive, not requiring specialized equipment and also using generally inexpensive polysaccharides. Finally, they look for interactions with polysaccharides, which in many cases are the relevant substrate for the enzyme under study. An important consideration, however, is discriminating between binding at the active site and a potential SBS or previously unrecognized CBM. Using active enzymes, as was done in this study, may help in this matter as catalysis might limit the contribution of the active site to the observed binding with these techniques. Competition with small molecules known to bind at the active site, may also help identify if binding is taking place elsewhere, however, caution is needed as these may also bind at a potential SBS or CBM. Comparison with other related enzymes known to contain an SBS may also provide important clues. Ultimately however, these methods are used to generate the hypothesis that an SBS is present, which then must be tested by other means. In this study we have taken that next step examining a group of six enzymes by SPR. The main drawback of SPR is that oligosaccharides must be used, which depending on the enzyme, may be quite expensive or unavailable, making it more appropriate for targeted studies, rather than screening. However, SPR has several advantages in the identification of SBSs. It uses relatively little protein and can examine a wide range of binding affinities compared to an alternative technique such as ITC. It is also able to be used with active enzymes as it measures equilibrium binding with a constant flow of ligand. Its most useful attribute, however, is that binding stoichiometry can be detected. This provides direct evidence of binding taking place outside of the active site, a critical consideration as will be discussed below.

### Positive Controls

Among the tested enzymes, three from GH13: GH13-1 [17,30,31], GH13-5 (PDB ID: 2E9B, unpublished) and GH13-6 [32,33] presented one or more SBSs in crystal structures and serve as positive controls for the purposes of this study. The properties of the two SBSs in GH13-1, barley α-amylase 1 (AMY1), have been extensively studied by using IPP [9,10], AE [9] and SPR assays [9,10]. However, exploitation of these techniques to detect SBSs in the absence of structural data has not been reported. In the previous studies of GH13-5 and GH13-6, the SBSs were not analyzed in depth, but SBSs studied by mutational analysis in human salivary α-amylase having a high degree of identity with porcine pancreatic α-amylase (GH13-6) [45–48], were found to be important for binding to and activity against starch [48]. SPR analysis indicates GH13-5 and 13-6 have multiple binding sites for β-cyclodextrin (Table 4), possibly representing the active site where both crystal structures show bound α-cyclodextrin [49], PDB ID: 2E8Z (unpublished), and one, perhaps more, SBSs. Noticeably, the affinity at these multiple sites are not distinguished from one another, suggesting that they have similar *K*_d_ values. This has been seen for other SBS containing enzymes, e.g. *Bacillus subtilis* [7] and *B*. *circulans* xylanases [50] and barley AMY1 (GH13-1) [9]. In xylanases, concurrence of active site and SBS binding affinity has been measured by NMR, SPR and ITC, using mutational analysis and active site inhibitors, indicating that the phenomenon was not an artefact of any particular technique. It may be that the affinity at SBSs and their cognate active sites have evolved in tandem to efficiently act together in substrate binding. As expected AMY1 binds to pullulan, amylopectin and glycogen in AE (its binding to amylose is just outside the cutoff chosen to define binding) and to starch granules by IPP. Neither GH13-5 that possesses a CBM48 nor GH13-6 binds to starch granules, but GH13-6 interacts with all α-1,4 linked glucan substrates and several other polysaccharides in AE (GH13-5 was not tested as it did not produce easily distinguishable bands). Binding of GH13-6 to non-substrates may result from the surface exposed nature of SBSs making them less selective in their interactions than active sites, which tend to bury a greater proportion of the substrates in a groove or tunnel in the protein.

The GH15-1 containing CBM20 interacts with all of the starch-like compounds in AE and additionally with galactan, whereas the version lacking CBM20 (GH15-2) does not. Thus binding to non-substrate polysaccharides may be a particular characteristic of SBSs as well as CBMs. While this phenomenon has not been studied before in SBS containing enzymes, it has been noted for CBMs. Recently Hernandez-Gomez et al. [51] examined the ability of crystalline cellulose specific CBMs to bind xyloglucan, a more soluble polymer thought to apply an entirely different mode of binding. They found that binding was weaker to xyloglucan than to crystalline cellulose, but still occurred on the same binding site. In another study, Strobel et al. [52] demonstrated that the cellulose binding face of a CBM1 was also binding to lignin. Furthermore, they attempted to increase the specificity for cellulose using a mutational approach, but as most mutants had reduced affinity for both cellulose and lignin, perhaps substantial overlap occurred among the binding sites for both ligands. In complex polysaccharide environments it can be advantageous for CBMs to attach onto other polysaccharides that are present in addition to the substrate of their appended enzyme. Mannanases are found with cellulose specific CBMs [53], thus targeting the far more abundant cellulose in the plant cell wall, while the active site maintains exquisite selectivity [39]. There may be evolutionary reasons for CBMs and SBSs to have lower specificity, which may go hand in hand with the highly exposed nature of these binding sites unavoidably resulting in this reduced specificity. In the CBM2 family there are both cellulose and xylan recognizing members, however it is possible to interconvert the specificity by mutation of a single residue [54]. This propensity for binding to non-substrate polysaccharides could prove useful in identifying enzymes possessing previously unrecognized CBMs or SBSs.

### Negative Controls

In complement to the positive controls analyzed, several proteins that have been extensively structurally characterized without detection of an SBS were included to serve as negative controls. The barley limit dextrinase (GH13-2) has been co-crystallized with several different ligands, recently including its natural substrate, an α-limit dextrin [2]. The only structure, however, which has shown binding outside of the active site was soaked with 400 mM maltotetraose. Soaking at lower concentrations did not result in detectable binding at this SBS, suspiciously located at a symmetry molecule interface, suggesting that it was an artefact. GH13-2, moreover, demonstrates no affinity in IPP and in AE only interacts with the non-substrate amylose. Similarly, crystal structures of barley β-amylase (GH14-1) in complex with different oligosaccharides did not reveal the presence of an SBS [34,35], moreover it does not bind any of the polysaccharides in AE and only to OSX by IPP. This enzyme does not exhibit binding to its substrate, starch, and since this is binding to a single polysaccharide, which is not biologically relevant, we conclude that this is most likely irrelevant non-specific binding. In addition, a β-mannanase (GH26-1) has crystal structures solved in complex with several oligosaccharides without showing an SBS and does not bind to any polysaccharides in the screening experiments.

GH48-1, a processive endoglucanase from *Clostridium cellulolyticum*, is a cellulosomal enzyme and hence part of a multi-enzyme assembly where it can take advantage of binding sites on other enzymes in the scaffoldin-enzyme complex [55]. Although crystal structures did not reveal any additional binding sites, this enzyme indeed is retarded by several polysaccharides in AE, including the non-substrates RHG and WAX; as expected GH48-1 binds to cellulose in IPP (Table 1). Further investigation shows that cellobiose, a potential inhibitor of binding, does not significantly suppress the interaction of GH48-1 with BBG in AE or in IPP with avicel. Cellopentaose, however, virtually eliminates GH48-1’s binding to avicel in IPP. Follow up by SPR reveals just one binding site for cellopentaose, which almost certainly is the active site as crystal structures show cellotetraose and cellohexaose accommodated in the active site [40]. Thus all GH48-1 interactions with cellulosic substrates seem mediated through the active site, while the possibility remains that binding to non-substrate polysaccharides takes place at an alternate location. This illustrates the point that while AE and IPP are very good for detecting the presence of binding sites, further experiments where the active site is blocked and/or the number of binding sites can be assessed are needed to rule out false positives of SBSs due to active site binding. There are a few reports on xylanases where the active site was blocked by covalent reaction with a mechanism-based inhibitor. Thus *B*. *circulans* xylanase treated with 2′,4′-dinitrophenyl 2-deoxy-2-fluoro-β-xylobioside ensured independent analysis of SBS binding in AE, while distinct active site data were achieved by mutation of the SBS [50]. Similarly, Cuyvers et al. [7] reacted the *B*. *subtilis* xylanase with the mechanism-based inhibitor 2,3-epoxypropyl β-D-xylopyranoside to investigate the SBS. Such inhibitors are absolutely useful tools in the study of non-catalytic site binding, but they are far from available for enzymes in general. Hence alternative, more universal strategies for detecting multiple binding sites, such as SPR are extremely valuable.

### Screening for Novel Binding Sites

Of the 28 enzymes not discussed already as positive or negative controls, 12 display binding to at least one ligand. These are GH5-1, GH6-1, GH8-1, GH10-1, GH11-1, GH13-3, GH31-1, GH53-1, GH62-1, GH77-1, GH84-1 and PL10-1. GH62-1 [18] and GH77-1 are currently undergoing further investigations and will not be discussed below. Additionally GH53-1, GH84-1 and PL10-1 each bind only to a single non-substrate polysaccharide that do not seem to be of biological relevance. Therefore we consider these to be spurious false positives and have not examined them further.

GH6-1, an endoglucanase from *Thermobifida fusca*, normally has a cellulose binding CBM2, which however, is omitted from the recombinant form used in this study to allow detection of one or more SBSs. It interacts with several polysaccharides, but most would be expected to serve as substrates or at least to bind in the active site (both KGM and XYG contain some unsubstituted β-1,4 linked glucose). Furthermore, SPR does not give evidence of multiple binding sites, in agreement with GH6-1 crystal structures of oligosaccharide complexes that showed binding only in the active site [56].

**Fig 3:**
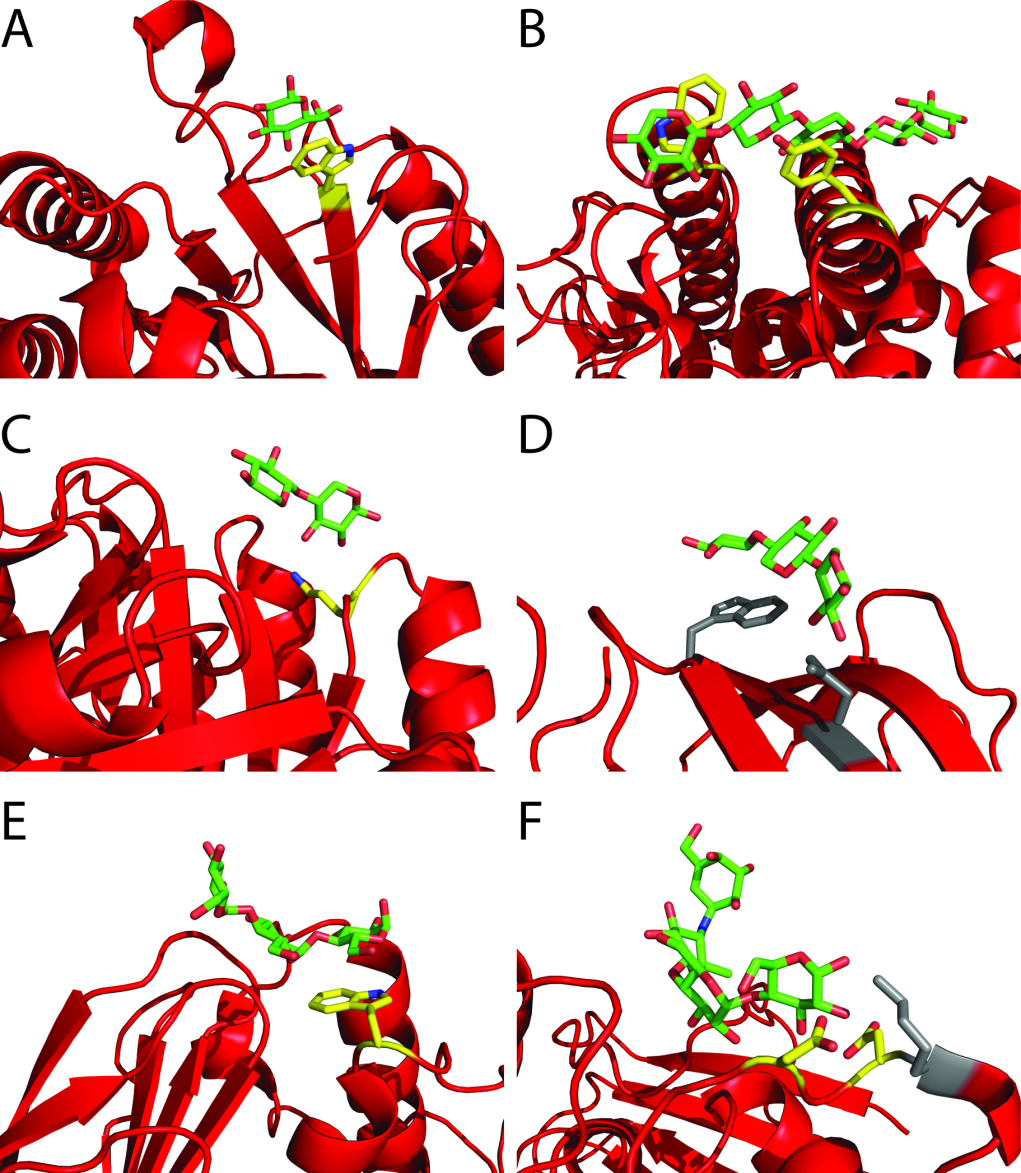
Conserved surface binding sites in related family members. The crystal structure is represented as a cartoon in red, with the bound carbohydratesshown in green (carbon) and red (oxygen). Conserved residues of the SBS also found in the enzymes in this studyare shown in yellow (carbon) and red (oxygen), while non-conserved residues are shown in grey. A shows the GH5 exo-β1—3 glucanase from *Candida albicans* (2PC8), B shows the GH8 xylanase from *Pseudoalteromonas haloplanktis* (2B4F), C shows the GH10 xylanase from *Thermoascus aurantiacus* (1GOQ), D shows the GH11 xylanase from Bacillus subtilis (2QZ3), E shows the GH13 (subfamily 5) α-amylase from *Bacillus* sp. 707, F shows the GH31 α-glucosidase from *Ruminococcus obeum* (3POC).

GH5-1 [57], GH8-1 [58], GH10-1 [59,60], GH11-1 [50,61], GH13-3 [62,63] and GH31-1 (PDB ID: 3NQQ, unpublished) all have a counterpart of the enzyme within the same family (or subfamily in the case of GH13-3) that has an SBS demonstrated by X-ray crystallography (see Fig 3). For GH5-1 and GH10-1 binding is observed to only a single ligand (Table 1) expected to be capable of binding at the active site. However, in both enzymes the residues comprising the SBS in the related family member are conserved. For GH5-1 this putative SBS would be located at W290, while for GH10-1, the alignment indicates a potential SBS primarily consisting of K38. This conservation does not necessarily indicate the presence of a functional SBS as changes in the surrounding residues could influence or even eliminate binding altogether as found for SBS2 of AMY1 (GH13-1). There is a second isoform AMY2 of this barley enzyme with high sequence identity to AMY1, including key residues in the two SBSs detected in AMY1. SBS2, however, seems non-functional in AMY2 [64], likely due the altered residues surrounding the conserved SBS2 binding residues [65]. Thus further testing would be needed to confirm the presence of an SBS in GH5-1 and GH10-1.

The xylanase GH11-1 interacts with five ligands (Table 1), including LAM and PUL which are not expected to be substrates. Three GH11 enzymes have been reported to possess an SBS, which occur, however, at two distinct structural locations [66]. None of these SBS residues are conserved within GH11-1. This does not necessarily indicate that GH11-1 has no SBS as there could be a third structural location for an SBS within GH11. Further investigation will be required to determine if GH11-1 indeed contains an SBS. In family GH13 there are several distinct structural locations for SBSs, which are often present within the same enzyme. For instance, the recently published structure of the *E*. *coli* branching enzyme demonstrated six different SBS positions occupied by linear maltooligosaccharides [67].

The *E*. *coli* α-amylase (GH13-3) is homologous to maltohexaose forming α-amylase from *Bacillus* sp. 707 known to have four SBSs [63]. Only one of these SBSs is conserved in GH13-3, which fits with the SPR β-cyclodextrin binding data yielding an average stoichiometry of 1.63, compatible with the presence of a second binding site beyond the active site. Based on alignment with the *Bacillus* enzyme, the location of the SBS is most likely centered at W346 in GH13-3.

Rice α-glucosidase (GH31-1) is the enzyme found to interact with the greatest variety of polysaccharides in both AE and IPP experiments. This may possibly be explained by the relatively wide variety of activities present within the GH31 family, including α-glucosidase, α-galactosidase, α-mannosidase and α-xylosidase, suggesting this family of enzymes is highly adaptable. Remarkably, residues comprising the SBS in *Blautia obeum* (formerly *Ruminococcus obeum*) GH31 α-glucosidase known to have an SBS are in part conserved in GH31-1 [68]. Somewhat unusually this SBS has no aromatic, but solely hydrogen bonding residues. Two of these, D651 and D653, are conserved in GH31-1 while a third, K654, is not. This in conjunction with the large variety of polysaccharides binding to GH31-1, suggests an SBS exists and most likely at a site equivalent to that in the *B*. *obeum* enzyme.

Family GH8 is multi-specific, containing xylanases, cellulases and chitinases among others. One enzyme, the xylanase from *Pseudoalteromonas haloplanktis* has been demonstrated by X-ray crystallography to possess an SBS [58] and its binding properties were explored in more detail [69]. GH8-1 is an endoglucanase from *Clostridium thermocellum* with the SBS from *P*. *haloplanktis* xylanase conserved (at W261 and Y315 in the *C*. *thermocellum* enzyme, see Fig 3). GH8-1 binds to several polysaccharides, including cellulose and xylan, and cellobiose suppresses the interaction with cellulose. SPR results in an average stoichiometry for cellopentaose of 2.33 suggesting the presence of two SBSs. While weak, there is also evident binding of xylotetraose and maltotetraose that probably reflects lower specificity of the SBS(s). While this is the first direct evidence of an SBS in a cellulase, both molecular dynamics [70] and crystalline cellulose binding experiments [71] previously indicated that binding outside the active site and the C-terminal CBM1 may occur in the *Trichoderma reesei* cellobiohydrolase Cel7A.

### Future Perspectives

AE, IPP and SPR have all been used in the past to study SBSs previously identified by structural studies. Here we demonstrate that these techniques are useful in detecting novel enzyme binding sites, also in the absence of a solved structure. Assessment of additional binding sites is not routine in characterization of new enzymes, however, thanks to these relatively simple tools, the presence of potential SBSs can be recognized fairly quickly. Indeed, the first known SBS was detected not in a crystal structure, but through techniques monitoring binding [72]. Localizing the binding site to particular residues is more challenging, however, besides structural techniques, chemical modification in the presence and absence ligands, coupled to proteolytic digestion and mass spectrometry analysis offers an alternative path. Indeed such differential labelling was used to identify an SBSs in barley α-amylase AMY2 [73].

SBSs appear to be fairly widespread in nature, but still relatively few studies examined their properties and importance to enzymes. Binding site screening techniques such as presented here can greatly expand knowledge about these important sites if employed more routinely in enzyme characterization. Additionally, while CBMs are easier to predict than SBSs based on sequences, there are still new CBM families to be discovered and characterized and these same techniques are equally suited for detecting binding by CBMs. However, interaction at the active site must always be kept in mind, although cases of binding to non-substrates or to insoluble substrates may indicate non-active site binding. Measuring the impact of non-substrate polysaccharides on enzyme activity against natural substrates provides one way of determining binding location. Follow up with a technique such as SPR that can determine the number of binding sites is an important verification step. Methods for selectively blocking the active site are of great utility, but are only available for relatively few enzymes. In this study we have demonstrated that AE, IPP and SPR are useful for identifying binding outside of the active site. Several of the examined enzymes very likely contain SBSs, including the first cellulase ever identified to possess an SBS. These techniques provide a simple toolbox for the routine identification of binding outside of the active site at SBSs and CBMs.

## Supporting Information

S1 File Supplementary Methods. (DOCX)

S2 File Supporting Data. All affinity electrophoresis data.
(XLSX)

S1 Table Primers used in this study. (DOCX)
