# Supplementary material for: Using Carbohydrate Interaction Assays to Reveal Novel Binding Sites in Carbohydrate Active Enzymes
Source: PLoS One. 2016 Aug 9;11(8):e0160112. doi: 10.1371/journal.pone.0160112 (PMC4978508; doi:10.1371/journal.pone.0160112)
Supplement: S1 File — (DOCX) [file pone.0160112.s001.docx]

**Supplementary Methods**

**Cloning**

**GH10-1, GH11-1 and AA09-1**

*Aspergillus nidulans* FGSC A4 was grown on minimal agar medium (0.6% NaNO3, 0.052% KCl, 0.052% MgSO4·7H2O, 1 mL trace element,12 mM sodium phosphate buffer pH 6.8, 1.25% agar; [1]) containing 1% oat spelt xylan at 30°C for 5 d. The spores were suspended in 0.02% TWEEN-80 and grown in minimal medium without agar at 30 °C for 1 d with shaking at 130 rpm. The cells were harvested by decanting the culture supernatant.

Two g of fungal cells was placed in a liquid nitrogen-cooled mortar with addition of liquid nitrogen and ground with a pestle until the cells became a fine powder. The mRNA isolation from the crushed fungal cells was performed using FastTrack 2.0 Kit for isolation of mRNA (Invitrogen) and the cDNA library was prepared using SuperScript III First Strand Synthesis System for reverse transcriptase polymerase chain reaction (Invitrogen). The *eglF* (AA09-1), *xlnA* (GH11-1) and *xlnC* (GH10-1) genes were obtained by amplification of target cDNA using Expand High Fidelity DNA polymerase (Roche, Basel, Switzerland) and oligonucleotide primers (Table S1), constructed based on the genomic sequence [2].

For AA09-1 the PCR product was digested by *Xho*I and *Xba*I (New England BioLabs) and cloned into the pPICZαA vector (Invitrogen). The plasmid was transformed into *Escherichia coli* DH5α (Invitrogen) and transformants were selected on low salt LB (1% tryptone, 0.5% yeast extract, 1% NaCl) supplemented with 25 μg/mL Zeocin. The purified plasmids (QIAgen plasmid midi kit; QIAgen, Hilden, Germany) were confirmed by full-length sequencing (Eurofins MWG Operon). The plasmids were linearized by *Pme*I and transformed into *Pichia pastoris* strain X-33 by electroporation (Micropulser; Bio-Rad Laboratories Inc, Hercules, CA). Transformants were selected on yeast peptone dextrose (1% yeast extract, 2% peptone, 2% dextrose) with 1 M sorbitol plate containing 100 µg/mL Zeocin at 30 °C for 3 days.

For both GH10-1 and GH11-1, PCR products were digested by *Nco*I and *Not*I (New England BioLabs) and cloned into the pET28-a(+) vector (Novagen, Merck KGaA, Darmstadt, Germany). The plasmids (pET28-a(+)-XlnA and pET28-a(+)-XlnC) were transformed into *E. coli* BL21(DE3) (New England BioLabs) by heatshock at 42 °C. Transformants were selected on LB supplemented with 30 μg/mL kanamycin and purified plasmids were confirmed by full sequencing (Eurofins MWG Operon).

**GH32-1 and GH32-2**

The *bfrA* (GH32-1; GenBank, gi: 58336834) and *scrB* (GH32-2; GenBank, gi: 58336737) were cloned by PCR from genomic DNA of *Lactobacillus acidophilus* NCFM (ATCC SD5221; Danisco USA Inc, Madison, WI, USA) grown in de Mann, Rogosa and Sharpe medium (Oxoid Ltd., Basingstoke, UK) under anaerobic conditions at 37 °C until late exponential phase. Cells were harvested by centrifugation, washed twice with phosphate-buffered saline, resuspended in lysis buffer (50 mM Tris-HCl, 1 mM EDTA, pH 8.0), and highly pure DNA was isolated as described [3]. Expand High Fidelity PCR System was used as DNA polymerase with oligonucleotide pairs constructed based on the genomic sequence [4]. bfrA-F and scrA-F (*Sac*I site is underlined) as 5´ forward primers for GH32-1 and GH32-2, respectively, and bfrA-R and scrA-R (*Hin*dIII site is underlined) as corresponding 3´ reverse primers (see Table S1). PCR products were purified (QIAquick Gel Extraction Kit; Qiagen), digested by *Sac*I and *Hin*dIII (New England Biolabs), and cloned into pCold I (Takara, Kyoto, Japan) resulting in expression plasmids *bfrA*/pCold I and *scrB*/pCold I propagated in *E*. *coli* DH5a (Novagen), purified (QIAprep Spin Miniprep Kit; Qiagen), and verified by sequencing.

**Protein Production and Purification**

**GH32-1 and GH32-2**

*E*. *coli* BL21(DE3) harbouring *bfrA*/pColdI and *scrB*/pColdI were grown at 12 ^o^C in LB medium containing 50 µg/mL ampicillin (6 × 1 L in 2 L shake flasks) and expression was induced by 0.1 mM isopropyl-1-thio-β-galactopyranoside (IPTG) at 12 ^o^C for 24 h. Cells were harvested (9000*g*, 10 min, 4 ^o^C), resuspended in 60 mL BugBuster Protein Extraction Reagents (Novagen) containing 2 µL Benzonate Nuclease (Novagen), incubated 30 min at room temperature, and centrifuged (19000*g*, 15 min, 4 ^o^C). The supernatant was filtered (0.45 µm) and applied to a HisTrap HP (5 mL; GE Healthcare) equilibrated with 20 mM HEPES pH 7.5, 0.5 M NaCl, 10 mM imidazole and washed with 20 mM HEPES pH 7.5, 0.5 M NaCl, 22 mM imidazole. Enzyme-containing fractions eluted by a linear 22−400 mM imidazole gradient in the same buffer were pooled, concentrated (Amicon Ultra Ultracel-10k; Millipore Corporation, Billerica, MA), and applied to HiLoad^TM^ 75 Superdex^TM^ 26/60 column (GE Healthcare) equilibrated with 10 mM MES pH 6.8, 0.15 M NaCl. Enzyme-containing fractions were pooled, dialyzed against 10 mM HEPES pH 7.0, and concentrated as above. All purification steps were performed at 4 ^o^C. Protein concentration was determined spectrophotometrically at 280 nm using *E*^0.1%^ = 2.14 (GH32-1) and 1.83 (GH32-2) determined by aid of amino acid analysis [5].

**GH10-1 and GH11-1**

*E. coli* BL21(DE3) harbouring GH10-1 and GH11-1 were grown in 1 L of LB containing 30 µg/mL kanamycin in 3 L plastic shake flask at 37°C to OD_600_ of 0.6. Expression was induced by 0.1 mM IPTG and continued at 12°C for 24 h. Cells were harvested (10,000*g*, 10 min, 4 °C) and resuspended in 30 mL BugBuster Protein Extraction Reagent containing 1 µL Benzonase Nuclease. Following 30 min incubation at 4 °C with rotating mixing, the supernatants were collected (16,000*g*, 4 °C, 20 min), filtered (45 µm) and applied to a 5 mL HisTrap HP column (GE Healthcare) equilibrated with 20 mM HEPES, 0.5 M NaCl, 10 mM imidazole, pH 7.5. After washing with 20 mM HEPES, 0.5 M NaCl, 22 mM imidazole, pH 7.5, followed by elution using a 22–400 mM imidazole linear gradient (flow rate: 1.0 mL/min 25 mL) fractions containing enzymes were pooled, concentrated (Centriprep YM10, Millipore), and applied to a HiLoad^TM^ 26/60 Superdex^TM^ G75 column (GE Healthcare) equilibrated with 20 mM HEPES, 0.15 M NaCl, pH 7.0 (flow rate: 0.5 mL/min). Fractions containing enzymes were pooled, concentrated, and buffer-exchanged to 20 mM HEPES pH 7.0. All purification steps were performed at 4 °C.

**AA09-1**

A single colony of *P. pastoris* harboring the gene for AA09-1 was used to inoculate 25 mL BMGY (1% yeast extract, 2% peptone, 100 mM postassium phosphate pH 6.0, 1.34% yeast nitrogen base, 4 x10^-5^% biotin, 1% glycerol) in a 250 mL sterile baffled flask. It was grown overnight at 30⁰C shaking at 200 rpm until it reached an OD_600_ of 2.5. This 25 mL culture was used to inoculate 1 L of BMGY in a 3 L sterile baffled flask. The culture was grown as above to OD_600_=3.

The cells were harvested in sterile centrifuge bottles at 3000*g* for 5 min at room temperature, then resuspended in 3 L BMMY (BMGY, but with the glycerol replaced with 0.5% methanol). These 3 L were divided into six 500 mL cultures each in 2.5 L sterile baffled flask. The baffled flasks were covered with two layers of cheesecloth which allows gas venting while preventing contamination. Such prepared cultures were incubated at 30 ⁰C and 180 rpm. The production was performed for three days, with methanol supplemented to a final concentration of 0.5% each day. The cells were harvested for 5 min at 3000*g* at room temperature. Culture supernatants containing the secreted protein were kept for purification.

The supernatants were concentrated to 150 mL and buffer exchanged into 25 mM MES pH 6 using a Pelicon ultrafiltration device (Millipore). This was then applied to a Capto^TM^ Q anion exchange column (GE Healthcare) which had been equilibrated with 25 mM MES pH 6 and then eluted with a linear gradient to 25 mM MES pH 6.5, 1 M NaCl. Eluted protein was then applied to a HiLoad^TM^ 26/60 Superdex^TM^ G75 gel filtration column equilibrated with 10 mM MES pH 6.5, 150 mM NaCl. The final purified protein was stored at 4 °C until needed.

**References**

[1] R.W. Barratt, G.B. Johnson, W.N. Ogata, Wild-type and mutant stocks of Aspergillus nidulans, Genetics 52 **(1965)** 233-246

[2] J.E. Galagan, S.E. Calvo, C. Cuomo, M. Li-Jun, J.R. Wortman, S. Batzoglou, S. Lee, Meray Bastürkmen, C.C. Spevak, J. Clutterbuck, V. Kapitonov, J. Jurka, C. Scazzocchio, M. Farman, J. Butler, Sequencing of Aspergillus nidulans and comparative analysis with A. fumigatus and A. oryzae, Nature 438 **(2005)** 1105-15

[3] J.H.A. Apajalahti, L. Särkilahti K., B. Mäki R.E., J.P. Heikkinen, P. Nurminen, W.E. Holben, Effective recovery of bacterial DNA and percent-guanine-plus-cytosine-based analysis of community structure in the gastrointestinal tract of broiler chickens, Appl.Environ.Microbiol. 64 **(1998)** 4084-4088

[4] E. Altermann, W.M. Russell, M. Azcarate-Peril, R. Barrangou, B.L. Buck, O. McAuliffe, N. Souther, A. Dobson, T. Duong, M. Callanan, S. Lick, A. Hamrick, R. Cano, T.R. Klaenhammer, Complete genome sequence of the probiotic lactic acid bacterium Lactobacillus acidophilus NCFM, Proc.Natl.Acad.Sci.U.S.A. 102 **(2005)** 3906-3912

[5] V. Barkholt, A.L. Jensen, Amino acid analysis: determination of cysteine plus half-cystine in proteins after hydrochloric acid hydrolysis with a disulfide compound as additive, Anal.Biochem. 177 **(1989)** 318-322
