# Supplementary material for: Using Carbohydrate Interaction Assays to Reveal Novel Binding Sites in Carbohydrate Active Enzymes
Source: PLoS One. 2016 Aug 9;11(8):e0160112. doi: 10.1371/journal.pone.0160112 (PMC4978508; doi:10.1371/journal.pone.0160112)
Supplement: S1 Table — (DOCX) [file pone.0160112.s003.docx]

S1 Table. Primers used in this study.

| **Name** | **Sequence** | **Purpose** |
| --- | --- | --- |
| bfrA-F | 5´-AAAGAGCTCAAGGCAAATACTTACTTATATAAAAAA-3´ | Forward to clone GH32-1 |
| bfrA-R | 5´-AAAAAGCTTTTACAAATCCACTTTTTTAAGTTCCATTC-3´ | Reverse to clone GH32-1 |
| scrA-F | 5´-AAAGAGCTCGAATGGACAAGAGAAAAACGTTATTTAC-3´ | Forward to clone GH32-2 |
| scrA-R | 5´-AAAAAGCTTTTAAAGAATTGTTTTCATATTCCAAAG-3´ | Reverse to clone GH32-2 |
| AA09-1-F | 5’- AATATTCTCGAGAAAAGAGAGGCTGAAGCTCACGGATACGTGACG-3’ | Forward to clone AA09-1 |
| AA09-1-R | 5’-AAATACTCT AGATTAACCACTGTACAGCTCAGGACCAGGCATAGGG-3’ | Reverse to clone AA09-1 |
| xlnC-F | 5’- TACATGCCATGGCCCAAAGCGCCAGCCTAAATGATCTT-3’ | Forward to clone GH10-1 |
| xlnC-R | 5’- AAGGAAAAAAGCGGCCGCAGACAGAGCGTTGACGATTGACG-3’ | Reverse to clone GH10-1 |
| xlnA-F | 5’-TACATGCCATGGCCACGCCCGTCGGGTCTGAAGACC-3’ | Forward to clone GH11-1 |
| xlnA-R | 5’- AAGGAAAAAAGCGGCCGCGTAAACAGTAATAGAAGCCGACCCAC-3’ | Reverse to clone GH11-1 |
